# Inspirational Women in Surgery: Professor Tahmina Banu, Bangladesh

**DOI:** 10.1007/s00268-022-06669-9

**Published:** 2022-07-27

**Authors:** Tanvir K. Chowdhury, Doruk Ozgediz

**Affiliations:** 1 Pediatric Surgery, Chittagong Medical College, Chittagong, Bangladesh; 2 UCSF Pediatric Surgery, San Francisco, USA

Professor Tahmina Banu was born in Sylhet, a north-eastern district in Bangladesh known for its natural beauty with hills and meadows.
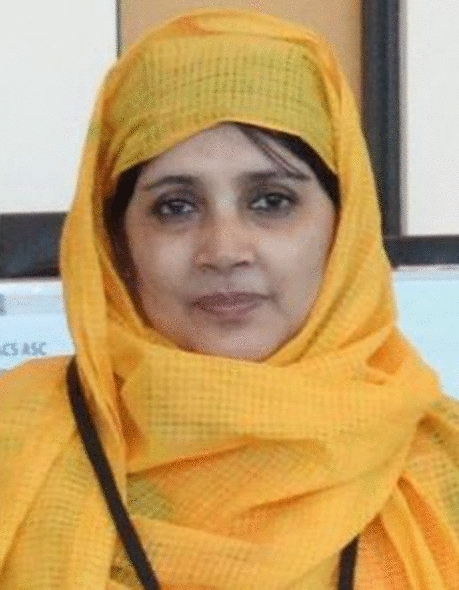
 She spent most of her childhood and her career in Chittagong, a port city by the Bay of Bengal and the commercial capital of the country. Her father, the Late Dr. AKM Abdul Matin, was a renowned pathologist in Chittagong, and her mother, the late Johara Begum, was a self-educated woman with invaluable life-experiences that enriched Tahmina’s career.

Tahmina attended Dr. Khastagir government girls’ high school and Chittagong College. She completed her basic medical degree (MBBS) from Chittagong Medical College and was in the first batch of students enrolled in the Pediatric Surgery post-graduate program (MS) from Dhaka Shishu (Children) Hospital which she completed in 1992. She was sent by the government to establish the Pediatric Surgery department in Chittagong Medical College Hospital (CMCH) in 1993. She took overseas training from Royal Children’s Hospital, Melbourne, the Women and Children’s Hospital in Adelaide, Australia, and from Long Island Jewish Medical center and Boston Children’s Hospital, USA, with the support of the Rowan Nicks Scholarship (1995), Australia, and Stephen L Gans Traveling Fellowship (1997), USA. She served in CMCH until 2016 and then established Chittagong Research Institute for Children Surgery (CRICS) in 2017. She also helped establish the “Dr. Matin Amrah Community Hospital” to provide surgical services to rural children, became an active member of the Global Initiative for Children’s Surgery in 2016, and the Healthy Bangladesh movement in 2017.

As the first female pediatric surgeon in Bangladesh, she is grateful for the mentorship she received from many medical professionals. However, the following mentors stand out for their profound guidance and wise counsel:

Professor ZM Chowdhury encouraged her to become a surgeon when there was no female surgeon in Bangladesh, and Professors AFM Masood, AFM Fazlul Karim and Sazzadur Rahman helped develop her skills and knowledge as a surgeon with hands-on training at different phases in her career.

Professor John M Hutson of Australia taught her to respect the dignity and privacy of children and encouraged her to conduct research and publications.

The opportunity to observe and assist Professor Alberto Pena and Professor Hardy Hendren—Tahmina described them surgical artists—from USA, instilled in her the confidence to manage challenging cases of complex, rare malformations with limited resources.

From the life of Prophet Muhammad (peace be upon him), Tahmina learned the value of compassion and dignity of all human beings and also the importance of striving against obstacles.

She admires Angela Merkel, the ex-chancellor of Germany, who inspired her with the ability to get things done in both normal and difficult times.

Tahmina chose a career in pediatric surgery because she believes health care, especially surgical care for children, is a form of social justice and equity.

Her most remarkable contribution to pediatric surgery has been the expansion and popularization of children’s surgery in Bangladesh, post-graduate program development (with over 70 pediatric surgeons trained), and development and implementation of a rural outreach surgical service for vulnerable populations [1]. She currently leads advocacy work to expand congenital anomaly detection and treatment globally with multiple leading child health organizations [2].

There have been many challenges along the way, including changing the stubborn mindset of the population and the medical community in Bangladesh about the need for children’s surgery, as well as gender bias and lack of assistance and support at the initial phase of her career. Her faith, determination, dedication, sincerity, and support from her siblings and the blessings of her parents helped her overcome adversities while remaining focused on her goals. Over time, her surgical successes and innovations travelled “mouth to mouth” and convinced and influenced the community, the print and digital media, and policymakers to support the development of children’s surgery. Her solid foundation of knowledge, technical skills, innovations, and “out of the box” thinking, and the ability to make quick decisions, helped her overcome many obstacles [3, 4]. She is known for her extraordinary bonds with her patients and their families.

She has successfully adopted the “whole society approach” to ensure affordable, timely and quality surgical care for those in need, particularly for poor children vulnerable to the dire effects of climate change and pandemics. She has been recognized regionally, nationally, and internationally for her accomplishments [5, 6].

She advised younger surgeons “There will always be challenges in life and these should be faced boldly. Use out of the box thinking to find solutions, believe in yourself, have a strong base of knowledge and skill, dedication, sincerity, and compassion for the patients, and this will certainly help to overcome adversity and difficult times. Lastly, women have the aptitude and capability of dealing with any challenges anywhere in the world.”
